# Pre-hospital emergency anaesthesia in 2025: ten years on from the modification of induction regimes

**DOI:** 10.1186/s13049-025-01354-x

**Published:** 2025-03-13

**Authors:** Mark Hodkinson, David Zideman, Kurtis Poole

**Affiliations:** Thames Valley Air Ambulance, Stokenchurch House, Oxford Road, Stokenchurch, HP14 3SX UK

**Keywords:** Pre-hospital emergency anaesthesia, Rapid sequence induction, Critical care team, Induction of anaesthesia, Pre-hospital emergency medicine

## Abstract

Pre-hospital emergency anaesthesia has become a common intervention in pre-hospital emergency medicine. Induction regimes have been modified significantly in the last decade largely guided by medical literature. A Thames Valley Air Ambulance working group have reviewed contemporary literature, developing updated guidelines on the induction and maintenance of pre-hospital emergency anaesthesia. The choice of agents remains unchanged, but there is a growing emphasis on providing a more tailored anaesthetic considering the patient’s frailty, background history and presenting physiology. Additional research into the optimal dose and combination of drugs is warranted, together with further exploration of patient’s physiological responses to pre-hospital anaesthesia.

## Background

Pre-hospital emergency anaesthesia (PHEA) is a complex intervention regularly performed by pre-hospital critical care teams in the United Kingdom (UK). Medical literature focuses predominately on delivering PHEA in trauma patients, demonstrating safe anaesthesia with high intubation success rates and few significant complications [1].

In the last ten years, evidence supporting the delivery of PHEA has grown extensively. Pre-hospital services have driven research considering patient characteristics, and induction and maintenance regimes. Some of the commonly quoted evidential studies may now be considered dated. Some studies, such as those by Sheridan and Perkins, question the benefits of PHEA in major trauma patients, citing sources from 2011–2017 [2]. The 2007 National Confidential Enquiry into Patient Outcome and Death (NCEPOD) reported that one in eight patients have an occluded airway on arrival at the emergency department [3]. This NCEPOD report made several recommendations, including the provision of airway management by pre-hospital teams that pre-date the development of major trauma networks in England [3]. More recently, Morton et al. reported on the journey of PHEA demonstrating that pre-hospital services are committed to safety and efficacy by the standardization of equipment, checklists and emergency drills [4]. Nevertheless, hypotension remains frequently reported and there are growing trends that associate this directly with induction regimes as the availability of data increases [5].

## Modification of induction regimes

In 2015, Lyon et al. proposed a significant modification to rapid sequence induction of anaesthesia in trauma patients [6], introducing a regime of fentanyl, ketamine and rocuronium. The regime was rapidly adopted across the UK [4]. The recommended regime was 3mcg/kg fentanyl, 2 mg/kg ketamine and 1 mg/kg rocuronium, with the modification to 0-1mcg/kg fentanyl, 1 mg/kg ketamine and 1 mg/kg rocuronium for hypotensive or unstable patients [6]. Although this regime was proven to be safe and effective [6], recent data links fentanyl to post induction hypotension [4]. The complexity of trauma patients and the increasing encounter of complex medical patients, such as those in the critical phase of recovery from out-of-hospital cardiac arrest, suggest that this regime may not be optimal for all patients. It is not clear that this regime is optimal for an increasingly diverse group of patients [5].

Post induction hypotension may not be solely attributable to fentanyl [4, 5], with multifactorial causes, particularly in major trauma patients with complex physiology confounded by haemorrhage or in the profoundly shocked medical case. This warrants further investigation. A recent retrospective cohort study highlighted the unreliability of non-invasive blood pressure in severe trauma or illness [7]. The increased use of arterial pressure monitoring in pre-hospital care is advantageous in aiding our understanding of the relationship between anaesthetic induction and post induction hemodynamic response, and should be considered in future research.

## A new era for induction regimes

In 2024, Thames Valley Air Ambulance (TVAA) established a working group to examine PHEA induction and maintenance regimes. A recent survey found that 27% of UK pre-hospital critical care teams utilize the fentanyl, ketamine, rocuronium regime described by Lyon et al. in 2015 [5, 6]. This demonstrates a shift in PHEA induction practice to a more considered anaesthetic regime, tailored to patient physiology, following the maturity of pre-hospital critical care services in the UK.

The TVAA working group summarized that although the choice of anaesthetic drugs remains at the discretion of the clinicians on scene, the decision as to which drugs to use and at what dose, should be based on a more detailed assessment of the patient’s clinical characteristics, including clinical frailty score, haemodynamic status and conscious level.

From September 2024, TVAA recommended a new adult regime that comprises a standardised dose of 100mcg fentanyl (where available), followed by an assessed dose of up to 2 mg/kg ketamine and a standardised dose of 100 mg rocuronium. A modified regime is available that omits the fentanyl and reduces the ketamine dose to up to 1 mg/kg, in patients who have a high frailty score, reduced conscious level or that have or are at risk of having post PHEA hypotension. In children, the recommended regime is 1-2 mg/kg ketamine and 1 mg/kg rocuronium. It was realised that not all inductions are delivered as pre-calculated doses in rapid sequence. The new regime emphasises the careful delivery of induction drugs akin to a delayed-sequence approach, monitoring both the conscious level and physiological responses prior to paralysis. It is recognised that there remains a requirement for rapid induction of anaesthesia for specific cases.

Post PHEA maintenance of anaesthesia by infusion is the recommended method at TVAA using either propofol (1-4 mg/kg/hr) or ketamine (1-2 mg/kg/hr), with analgesia (opiate or ketamine bolus) and repeat paralysis (0.5 mg/kg rocuronium) every 30–40 min, as required. The physiological parameters to monitor depth and adequacy of anaesthesia are defined as no tearing and a reduction in any tachycardia and associated hypertension. Although not absolute, these parameters have been found to guide clinicians in maintaining anaesthesia [2].

There are limitations in encouraging regimes guided by clinician discretion and assessment of patient factors. Firstly, it can be challenging to objectively identify level of consciousness and deliver optimal titration. Secondly, information on co-morbidities and frailty may not be available. Thirdly, reducing fentanyl doses may not obtund vagal response during laryngoscopy. Finally, standard regimes such as a 3:2:1 minimise the requirement for additional bandwidth on critical care teams.

These limitations are mitigated by expert experience amongst PHEA practitioners, increased exposure to diverse patient cohorts and the availability of invasive blood pressure monitoring. Our established clinical governance system closely monitors every PHEA case, including specific review of regime and efficacy, providing direct clinician feedback. It remains too early to analyse the effects of these new regimes on patient outcomes. Outside of survey data [5], and to our knowledge, this is the first documented transition away from previously accepted standardised induction regimes.

## Conclusion

The choice of induction regime and the patient cohort in PHEA practice has diversified with growing experience in pre-hospital settings. Establishing an induction regime framework based on clinical experience and patient-specific factors that de-emphasises fentanyl may maintain efficacy and reduce complications such as hypotension. Current literature often focuses on individual services or regions warranting national-level investigation to audit PHEA practice and patient outcomes.

## Data Availability

No datasets were generated or analysed during the current study.

## Abbreviations

PHEA: Pre-Hospital Emergency Anaesthesia
UK: United Kingdom of Great Britain & Northern Ireland
NCEPOD: National Confidential Enquiry into Patient Outcome & Death
TVAA: Thames Valley Air Ambulance
